# Cost-effectiveness analysis of elbasvir-grazoprevir regimen for treating hepatitis C virus genotype 1 infection in stage 4-5 chronic kidney disease patients in France

**DOI:** 10.1371/journal.pone.0194329

**Published:** 2018-03-15

**Authors:** Franck Maunoury, Aurore Clément, Chizoba Nwankwo, Laurie Levy-Bachelot, Armand Abergel, Vincent Di Martino, Eric Thervet, Isabelle Durand-Zaleski

**Affiliations:** 1 Statesia, Le Mans, France; 2 MSD FRANCE, Paris, France; 3 Merck & Co., Inc., Kenilworth, New Jersey, United States of America; 4 Hepato-gastro enterology Service, CHU Estaing, Clermont-Ferrand, France; 5 Hepatology Department, Franche-Comté University and Besançon University hospital, Besançon, France; 6 HYPPARC Department, Nephrology Service, Paris Descartes University, Paris, France; 7 Georges Pompidou European Hospital (ET), Paris, France; 8 Ile-De-France URC ECO, Department of Public Health, Henri Mondor Hospital, Créteil, France; University of North Carolina at Chapel Hill School of Medicine, UNITED STATES

## Abstract

**Objective:**

To assess the cost-effectiveness of the elbasvir/grazoprevir (EBR/GZR) regimen in patients with genotype 1 chronic hepatitis C virus (HCV) infection with severe and end-stage renal disease compared to no treatment.

**Design:**

This study uses a health economic model to estimate the cost-effectiveness of treating previously untreated and treatment experienced chronic hepatitis C patients who have severe and end stage renal disease with the elbasvir-grazoprevir regimen versus no treatment in the French context. The lifetime homogeneous markovian model comprises of forty combined health states including hepatitis C virus and chronic kidney disease. The model parameters were from a multicentre randomized controlled trial, ANRS CO22 HEPATHER French cohort and literature. 1000 Monte Carlo simulations of patient health states for each treatment strategy are used for probabilistic sensitivity analysis and 95% confidence intervals calculations. The results were expressed in cost per quality-adjusted life year (QALY) gained.

**Patients:**

The mean age of patients in the HEPATHER French cohort was 59.6 years and 56% of them were men. 22.3% of patients had a F0 fibrosis stage (no fibrosis), 24.1% a F1 stage (portal fibrosis without septa), 7.1% a F2 stage (portal fibrosis with few septa), 21.4% a F3 stage (numerous septa without fibrosis) and 25% a F4 fibrosis stage (compensated cirrhosis). Among these HCV genotype 1 patients, 30% had severe renal impairment stage 4, 33% had a severe renal insufficiency stage 5 and 37% had terminal severe renal impairment stage 5 treated by dialysis.

**Intervention:**

Fixed-dose combination of direct-acting antiviral agents elbasvir and grazoprevir compared to no-treatment.

**Results:**

EBR/GZR increased the number of life years (6.3 years) compared to no treatment (5.1 years) on a lifetime horizon. The total number of QALYs was higher for the new treatment because of better utility on health conditions (6.2 versus 3.7 QALYs). The incremental cost-utility ratio (ICUR) was of €15,212 per QALY gained for the base case analysis.

**Conclusions:**

This cost-utility model is an innovative approach that simultaneously looks at the disease evolution of chronic hepatitis C and chronic kidney disease. EBR/GZR without interferon and ribavirin, produced the greatest benefit in terms of life expectancy and quality-adjusted life years (QALY) in treatment-naïve or experienced patients with chronic hepatitis C genotype 1 and stage 4–5 chronic kidney disease including dialysis patients. Based on shape of the acceptability curve, EBR/GZR can be considered cost-effective at a willingness to pay of €20,000 /QALY for patients with renal insufficiency with severe and end-stage renal disease compared to no treatment.

## Introduction

Chronic hepatitis C is a liver disease caused by the hepatitis C virus (HCV) which can lead to cirrhosis in 10–20% of cases, in the absence of treatment at a median time of 10 to 30 years or even hepatocellular carcinoma (HCC) with an annual incidence of 1 to 5% in patients with cirrhosis. In France, genotype 1 is the most common (61%). Hepatitis C is more common in patients with renal disease than in the general population due to nosocomial transmission during dialysis or blood transfusion that occurred before 1994 [1]. A French study published in 2011 [2], conducted among 4,718 patients in 56 dialysis centers estimated the overall prevalence of 7.7% among hemodialysis patients (95% confidence interval of [6.9; 8.5]). In patients with concomitant chronic renal failure and chronic hepatitis C, there is an increased risk of all-cause mortality and mortality from liver disease. This increased risk is associated with the negative impact of HCV on renal function. Kidney disease is the cause of the most common mortality in patients with HCV: The mortality at 10 years in patients with renal disease is between 33 and 49% [3, 4].

The main purpose of the HCV treatment is to achieve sustained virological response (SVR), defined as undetectable HCV ribonucleic acid (RNA) 12 weeks after end of treatment. This viral eradication is seen as a valid marker of virological cure correlated with reduction in all-cause mortality [5].

Elbasvir (EBR, NS5A inhibitor)/grazoprevir (GZR, NS3/4A protease inhibitor) differs from some other direct acting antiviral because of its extra renal clearance (>90%) and a different profile in terms of drug interaction. The fixed dose combination regimen of EBR/GZR for 12 weeks as a single daily dose was recently found to be safe and achieved high rates of SVR across the CKD patient subgroups included in the study in a multicenter randomized phase 2/3 double-blind, placebo controlled clinical trial (C-SURFER trial [6],www.clinicaltrials.gov NCT02092350), consisting of 235 treatment naïve or experienced chronic HCV genotype 1 patients with chronic kidney disease stages 4 and 5, including patients undergoing hemodialysis, with or without cirrhosis.

Our objective was to assess the cost-effectiveness 12 weeks of EBR/GZR in the treatment of patients infected with chronic HCV genotype 1 and chronic renal failure stage 4 or 5 (creatinine clearance <30 mL / min / 1.73 m^2^) including patients on dialysis.

## Methods

The analysis was based upon a cost-utility HCV-CKD model which included both medical and economic criteria from a collective (“all payers”) perspective.

### Treatment comparators

The clinical and economic impact of using EBR/GZR was compared to no treatment. The rationale for this choice was based on 1) the latest recommendations of the French Association for Liver study AFEF [7] in 2015 in which EBR/GZR regimen is the only recommended treatment for stage 4–5 CKD and HCV genotype 1 patients; and 2) the lack of robust clinical data assessing the efficacy and safety for other direct-acting antivirals without interferon and ribavirin in this subpopulation of HCV patients. French clinical experts have validated the "no treatment strategy" as standard of care, based on the June 2015 recommendations [7] updated in February 2016 by AFEF and the latest communications at AASLD congress in November 2015. In genotype 1 patients with a creatinine clearance <30 ml / min / 1.73m2, treatment with elbasvir-grazoprevir for 12 weeks without ribavirin is the recommended regimen since June 2015 according to the recommendations of the AFEF [7]. The updated February 2016 recommendations issued by AFEF [4] confirm, with a high level of evidence, the role of elbasvir-grazoprevir as a reference treatment in HCV genotype 1 patients with severe or terminal renal insufficiency.

In the core model (US model) [8], efficacy of pegylated interferon (peg-IFN) [8,9,10] and pegylated interferon plus ribavirin (peg-IFN/RBV) was based on the results of a meta-analysis [11,12]. We did not consider peg-IFN/RBV because for patients with CKD 4–5 stages and kidney transplant this regimen is not tolerated and other treatments are not available. This is validated by French clinical experts.

### Model overview: A lifetime homogeneous Markov model

The modeling approach complied with the French National Authority for Health (Haute Autorité de Santé—HAS) and the CHEERS guidelines [13,14]. The lifetime homogeneous Markov model structure was based on observed data from the C-SURFER trial and patient characteristics from the HEPATHER French cohort “Therapeutic options for hepatitis B and C: a French cohort” which is a national multicenter prospective observational cohort study of subjects with past or present viral hepatitis B or C (ClinicalTrials.gov, number NCT01953458). The C-SURFER trial and the HEPATHER French cohort received ethical approval. The C-SURFER clinical trial was done at 68 centres in the USA, Argentina, Australia, Canada, Estonia, France, Israel, South Korea, Lithuania, Netherlands, Spain, and Sweden in accordance with the Declaration of Helsinki, the International Conference on Harmonization guidelines, and other regulations governing clinical study conduct. The protocol was approved by an independent ethics committee or institutional review board at each participating site. All patients provided written informed consent. Written informed consent has been obtained too from each patient before enrollment in the French HEPATHER cohort. The protocol is conducted in accordance with the Declaration of Helsinki and French law for biomedical research and approved by the "CPP Ile de France 3" Ethics Ccommittee (Agreement Number: 2943). The cohort is conducted in agreement with the Loi informatique et libertés (January 6, 1978, modified by the July 1, 1994 law and finalized by the August 6, 2004 law).

The model was programmed using Visual Basic Application with the Excel^®^ 2007 software. The chronic HCV and chronic kidney disease (CKD) model is a discrete-time, state-transition Markov model programmed in Microsoft Excel^®^ (Microsoft Corp., Redmond, WA). The model was fully parameterized to run the base case and sensitivity analyses. One-way deterministic sensitivity analysis (DSA) and probabilistic sensitivity analysis (PSA) were implemented Visual Basic macros. The model combined major complications of both CKD and liver diseases. The health states for CKD were defined following the National Kidney Foundation Kidney Disease Outcomes Quality Initiative (NKF KDOQI) guidelines which are based on kidney damage or glomerular filtration rate (GFR). To take into account the increased risk of developing end-stage renal disease (ESRD) or death from HCV infection among CKD patients, we adjusted the baseline CKD progression probabilities and other-cause mortality using hazard rates obtained by comparing progression and mortality rates among HCV infected with rates among patients without HCV.

### Model description

The HCV model is a multi-cohort Markov model that simulated each cohort using the natural history of progression of HCV disease through a lifetime horizon. The structure of HCV and liver complications model was based on previously published and validated Markov cohort model [8,9,10].

**Fig 1:**
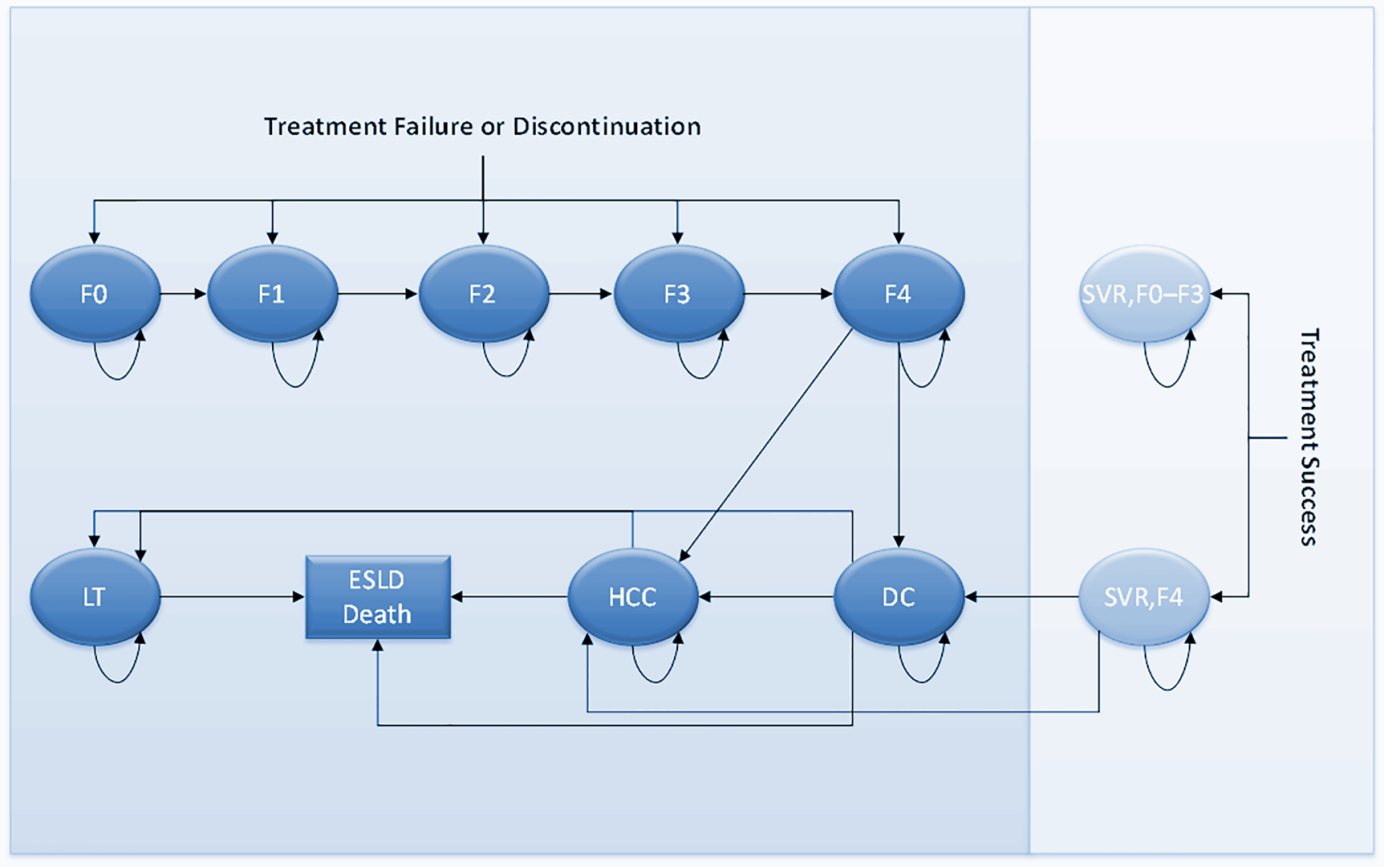
State-transition diagram for chronic hepatitis C and liver disease model. ESLD: End-Stage Liver Disease, F0: No fibrosis, F1: Portal fibrosis without septa, F2: Portal fibrosis with few septa, F3: Numerous septa without fibrosis, F4: Compensated cirrhosis, DC: Decompensated cirrhosis, HCC: Hepatocellular carcinoma, LT: Liver transplant, SVR, F0–F3 and SVR, F4: Patient’s baseline fibrosis stage before treatment and achievement of sustained virologic response (SVR).

Each cohort was determined by the following risk factors or demographic characteristics: age, sex (male/female), and baseline fibrosis score (F0–F4). The model’s structure is based on the following 10 health states: no fibrosis (F0), portal fibrosis without septa (F1), portal fibrosis with few septa (F2), numerous septa without fibrosis (F3), compensated cirrhosis (F4), decompensated cirrhosis (DC), hepatocellular carcinoma (HCC), liver transplant (LT), and patient’s baseline fibrosis stage before treatment and achievement of sustained virological response (“SVR, F0–F3” and “SVR, F4”) (Fig 1).

An SVR is considered a cure for patients who were originally non-cirrhotic (i.e., baseline fibrosis score of F0, F1, F2 or F3). Previously cirrhotic patients (i.e., baseline fibrosis score of F4) are assumed to have an excess risk of DC and HCC even if they achieved SVR. Thus, the SVR states are collapsed into 2 major groups (F0-F3,F4) instead of 5 health states (F0,F1,F2,F3,F4).

Progression to DC only occurs in cirrhotic patients (health states of compensated cirrhosis), and the health state DC which consists of multiple outcomes (i.e., ascites, variceal hemorrhage, and encephalopathy) is aggregated into one. Moreover, the DC, HCC, and liver LT states were not separated into two states (for example, DC and post DC) as was done in some recent models [8,9,10] so as to account more accurately for different mortality rates, costs or utilities of DC, HCC, and LT during the first year and subsequent years. However, several old and recent studies shared similar streamlined HCV model’s structure where each of these health states is aggregated into only one state. Examples include Najafzadeh et al [15], McEwan et al [16], Gissel et al [17], Leleu et al [18], Younossi [19], and Zhang et al [20]. Progression to HCC only occurs in cirrhotic patients (health states of compensated or decompensated cirrhosis). This excludes the risk of HCC even among patients with the F3 state. This assumption seems acceptable considering the very low frequency of this transition [21]. Patients in chronic HCV health states (including the states of mild HCV F0 and F1) cannot spontaneously clear HCV. There is no progression to more severe health states (i.e., cirrhosis) during therapy or subsequent follow-up for patients who respond to treatment. Patients who do not respond to therapy can progress to more severe health states during therapy or subsequent follow-up. An SVR is considered a cure for patients who were originally non-cirrhotic (i.e., baseline fibrosis score of F0, F1, F2 or F3). These patients are also assumed to be at no risk for reactivation of HCV infection or re-infection with HCV. Previously cirrhotic patients (i.e., baseline fibrosis score of F4) are assumed to have an excess risk of DC and HCC even if they achieved SVR. Thus, the SVR states are collapsed into 2 major groups (F0-F3,F4) instead of 5 health states (F0,..F4). It’s the reason why regression of fibrosis post-SVR is not modeled.

**Fig 2:**
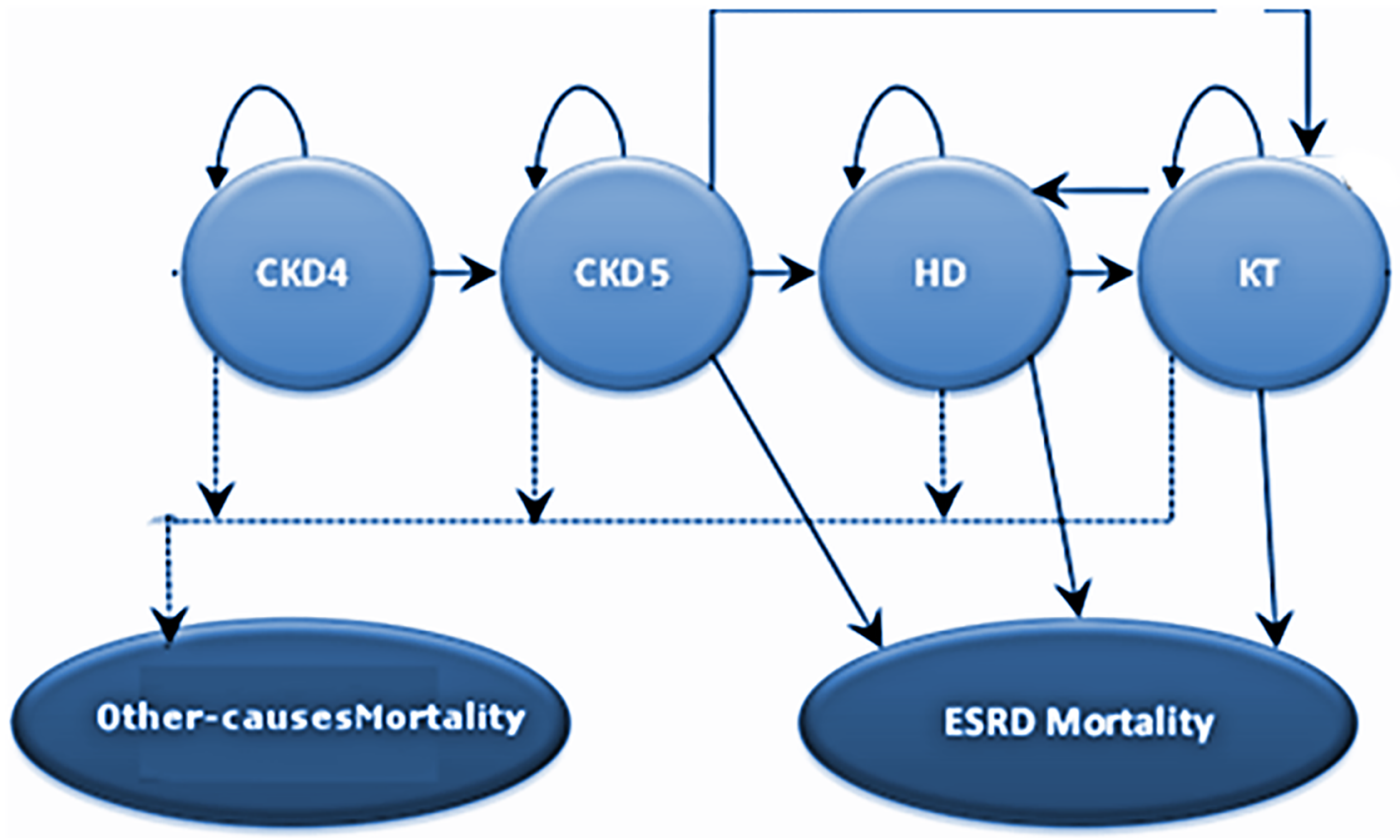
Simplified state-transition diagram for the chronic kidney disease (CKD) model. The health states for CKD were defined following the National Kidney Foundation K/DOQI guidelines. CKD stages considered in the model are: CKD4 (GFR 15–29 ml/min/ 1.73 m2), CKD5 (GFR < 15 ml/min/ 1.73 m2). In addition, we also include separate health states for hemodialysis (HD) and kidney transplantation (KT).

The CKD structure of the combined model builds on the framework of previous models [22,23,24,25,26,27] (Fig 2).

The health states for CKD were defined following the National Kidney Foundation K/DOQI guidelines. CKD stages considered in the model are: CKD4 (GFR 15–29 ml/min/ 1.73 m2), CKD5 (GFR < 15 ml/min/ 1.73 m2). In addition, we also include separate health states for hemodialysis (HD) and kidney transplantation (KT).

The combined model consists of 40 mutually exclusive states (Table 1) representing CKD status (CKD4, CKD5, HD, and KT) and liver disease conditions (SVR-F0–F3; SVR-F4, F0, F1, F2, F3, F4, DC, HCC, and LT). In addition, it tracks separately three types of mortality: ESLD mortality, ESRD mortality, and other-causes mortality. The model used an annual Markov cycle to predict the incidence and progression of CKD and liver diseases and related complications in a cohort of patients stratified by the following baseline characteristics: sex, age, liver fibrosis status, and CKD status.

**Table 1:** 40 mutually exclusive health states of the HCV-CKD combined model.

1	SVR-F0-F3, CKD4	9	F0,CKD4	17	F2,CKD4	25	F4,CKD4	33	HCC,CKD4
2	SVR-F0-F3, CKD5	10	F0,CKD5	18	F2,CKD5	26	F4,CKD5	34	HCC,CKD5
3	SVR-F0-F3,HD	11	F0,HD	19	F2,HD	27	F4,HD	35	HCC,HD
4	SVR-F0-F3,KT	12	F0,KT	20	F2,KT	28	F4,KT	36	HCC,KT
5	SVR-F4,CKD4	13	F1,CKD4	21	F3,CKD4	29	DC,CKD4	37	LT,CKD4
6	SVR-F4,CKD5	14	F1,CKD5	22	F3,CKD5	30	DC,CKD5	38	LT,CKD5
7	SVR-F4,HD	15	F1,HD	23	F3,HD	31	DC,HD	39	LT,HD
8	SVR-F4,KT	16	F1,KT	24	F3,KT	32	DC,KT	40	LT,KT

The cohort of patients at the model entry was defined by age and sex and distributed according to CKD 4–5 stages, hemodialysis treatment, and hepatic fibrosis stages previously defined (F0, F1, F2, F3, and F4). The renal insufficient stage 4 (severe renal insufficiency) or 5 (end-stage renal insufficiency) patients are supposed to be treated for chronic hepatitis C at the beginning of the simulation. The results of sustained virological response (SVR) of EBR/GZR treatment were applied to the treated patients and set as null for the strategy “No HCV treatment”. The Markov states “ESLD Mortality”, “ESRD Mortality”, and “Other-causes Mortality” are absorbent states. In each health state a transition towards the state “Other-causes Mortality” is possible. “ESRD Mortality” health state was assigned to the CKD5, hemodialysis (HD) or kidney transplantation (KT) health states only.

### Baseline population characteristics

To generate the data of the simulated population in the French context, a study was done in partnership with the ANRS (France REcherche Nord&sud Sida-vih Hépatites), INSERM transfert (Institut national de la santé et de la recherche médicale) and MSD, using an extraction of the ANRS CO22 HEPATHER database in February 5, 2016, on patients included in the HEPATHER cohort. The statistical analysis involved 99 patients and showed no significant difference at the 5% level regardless the genotype 1 and genotype 4 patient characteristics. Patient characteristics are described in Table 2. The mean age of these French HCV-CKD patients was of 59.6 years old and 56% of them were men. 22.3% of patients had a F0 fibrosis stage (no fibrosis), 24.1% a F1 stage (portal fibrosis without septa), 7.1% a F2 stage (portal fibrosis with few septa), 21.4% a F3 stage (numerous septa without fibrosis) and 25% a F4 fibrosis stage (compensated cirrhosis). Among these HCV genotype 1 patients, 30% had severe renal impairment stage 4, 33% a severe renal insufficiency stage 5 and 37% with terminal severe renal impairment stage 5 treated by dialysis. The initial cohort was assigned to CKD4, CKD5 and CKD5 with dialysis health states combined to hepatic fibrosis stage of the patient (F0 to F4) according to the proportion of renal insufficient patients with hepatitis C at stage F0, F1, F2, F3 or F4.

**Table 2:** Simulated population characteristics for the base case analysis (HEPATHER HCV-CKD population).

Item	Value *
Age at baseline (years)	59.6
% Male at baseline	56%
Baseline distribution of patients according to fibrosis stage	
F0	0.223
F1	0.241
F2	0.071
F3	0.214
F4 (compensated cirrhosis)	0.250
Baseline distribution of patients according to kidney disease stage	
CKD4 (15 < GFR ≤ 30 ml/min/1.73m2)	0.300
CKD5 (GFR ≤ 15 ml/min/1.73m2)	0.330
CKD5 treated by dialysis	0.370

*Source.Reference—HEPATHER

### Model inputs and transition probabilities

HCV model parameters were obtained from previous published studies and French registries (Table 3). CKD model inputs were derived from a targeted review of the published literature (Table 4).

**Table 3:** Liver disease model inputs.

Parameters	Baseline	Range	Reference
Annual transition probability			
Fibrosis Progression			
F0 to F1	0.072	0.068–0.076	[28]
F1 to F2	0.101	0.098–0.103	[28]
F2 to F3	0.108	0.106–0.111	[28]
F3 to F4 (compensated cirrhosis)	0.210	0.206–0.213	[28]
F4 to DC (decompensated cirrhosis)	0.050	0.017–0.100	[28]
F4 to HCC (hepatocellular carcinoma)	0.036	0.020–0.056	[28]
DC to HCC	0.036	0.020–0.056	[28]
SVR, F4 to DC	0.004	0.002–0.011	[28]
SVR, F4 to HCC	0.010	0.005–0.011	[28]
Probability of Receiving a Liver Transplant			
DC	0.120	0.097–0.145	[28]
HCC	0.170	0.143–0.199	[28]
Probability of death due to liver disease			
DC 1st year to death*	0.390	0.200–0.550	[28]
DC following years to death*	0.125	0.077–0.182	[28]
DC average 1st year and following years to death	0.130	0.125–0.390	[18,28]
HCC 1st year to death*	0.540	0.503–0.577	[28]
HCC following years to death*	0.270	0.238–0.303	[28]
HCC average 1st year and following years to death	0.430	0.270–0.540	[18,28]
LT Liver transplantation 1st year to death*	0.160	0.147–0.173	[28]
LT Liver transplantation following years to death*	0.032	0.026–0.039	[28]
LT average 1st year and following years to death	0.060	0.032–0.160	[18,28]

SVR—sustained virologic response; F0—no fibrosis; F1 –portal fibrosis without septa; F2 –portal fibrosis with few septa; F3 –numerous septa without cirrhosis; F4 –cirrhosis; DC—decompensated cirrhosis; HCC—hepatocellular carcinoma.

*These data allowed the calculation of weighted mean utilities and costs balanced between utilities and costs for DC, HCC, LT 1st year states and DC/HCC/LT following years states. Calculation was as follows: These probabilities 1st year and following years also made it possible to estimate the lower and upper probability values for ESLD-related death around the average values (1st year and following years) to advanced DC/HCC/LT states published by Leleu *et al*. 2015 [19].

**Table 4:** CKD model inputs.

Parameters	Baseline	Range	Reference
Annual transition probability			
CKD4 to CKD5	0.081	0.067–0.096	[27]
CKD5 to HD	0.434	0.430–0.439	[29]
CKD5 to KT	0.035	0.034–0.037	[29]
HD to KT	0.048	0.046–0.050	[29]
KT to HD	0.086	0.084–0.088	[29]
CKD5 to death	0.070	0.068–0.072	[29]
HD to death	0.125	0.123–0.128	[29]
KT to death*	0.029	0.027–0.030	[29]
Hazard rates			
Risk of progression of CKD given HCV (all stages)	1.70	1.2–2.4	[30]
Risk of MI and stroke (CKD stage 4)	2.80	2.6–2.9	[31]
Risk of MI and stroke (CKD stage 5)	3.40	3.1–3.8	[31]
Risk of all-cause mortality (CKD stage 4)	3.20	3.1–3.4	[31]
Risk of all-cause mortality ESRD (CKD stage 5, HD)	5.90	5.4–6.5	[31]

CKD, Chronic Kidney Disease; HD, Haemodialysis; KT, Kidney Transplant; HCV, Hepatitis C Virus; MI, Myocardial Infarction; ESRD, End-Stage Renal Disease.

*This data allowed estimating the weighted annual mean cost for kidney transplant.

### Clinical efficacy

Clinical data source was obtained from the multicentre randomized-controlled C-Surfer study [6] which compared the impact of EBR/GZR regimen and of no-treatment option on the SVR rate. In the pre-specified primary population, the proportion of patients achieving SVR 12 weeks after the completion of therapy was 99% (115/116).

### Utilities

The baseline analysis was a cost-utility analysis. Only the utilities related to chronic hepatitis C were used in the base case analysis.

A literature review found no data on utilities of the combined health states related to the concomitance of chronic hepatitis C and chronic kidney disease. The quality of life data from the C-SURFER clinical trial [6] did not use a validated utility score. We considered only utility values for chronic hepatitis C with a sensitivity analysis to estimate the impact of utility decrements associated with CKD [32].

Disutility related to the anti-HCV treatments was not considered.

The utility values are detailed in Table 5.

**Table 5:** Utility values for Natural History Health States, chronic hepatitis C.

Health States	Utilities			Source/Reference
	Average	Lower limit (95% average)	Upper limit (105% average)	
SVR. F0 mild chronic hepatitis C	1.00	1.00	1.00	Assumption
SVR. F1 mild chronic hepatitis C	1.00	1.00	1.00	Assumption
SVR. F2 moderate chronic hepatitis C	1.00	1.00	1.00	Assumption
SVR. F3 moderate chronic hepatitis C	1.00	1.00	1.00	Assumption
SVR. F4 compensated cirrhosis	1.00	1.00	1.00	Assumption
F0 mild Chronic hepatitis C	0.82	0.78	0.86	[30]
F1 Mild chronic hepatitis C	0.82	0.78	0.86	[30]
F2 Moderate chronic hepatitis C	0.78	0.74	0.82	[30]
F3 Moderate chronic hepatitis C	0.67	0.61	0.73	[30]
F4 Compensated cirrhosis	0.67	0.61	0.73	[30]
Decompensated cirrhosis 1st year	0.51	0.37	0.65	[30], assumption
Decompensated cirrhosis following years	0.51	0.37	0.65	[30]
Hepatocellular carcinoma 1st year	0.51	0.37	0.65	[30]
Hepatocellular carcinoma following years	0.51	0.48	0.54	[30]
Liver transplantation 1st year*	0.46	0.44	0.48	[30]
Liver transplantation following years*	0.80	0.76	0.84	[30]
Liver transplantation (weighted mean value for the first and subsequent years)	0.75	0.53	0.91	[30], calculation*
Death	0.00	0.00	0.00	[33]
Hemodialysis	0.44	0.33	1.00	[30]
Kidney transplant	0.71	0.53	1.00	[30]

*These utility data allowed estimating the weighted mean utilities for LT 1st year and LT following years. The calculation is as follow: Weighted mean utility for LT = annual utility for LT 1^st^ year x annual transition probability from LT 1^st^ year to death + annual utility for LT following years x (1—annual transition probability from LT 1^st^ year to death).

The assumption that all patients achieving SVR have a quality of life (QoL) of 1.00 could be challenged. Different studies have shown a slight increase in QoL, but without reaching “perfect health” [30,31]. Considering these references, we performed a scenario analysis considering a 0.82 utility index for SVR state, whatever the fibrosis stage. This value is equal to the utility index used for F0-F1 health states. For this scenario, the ICUR increases at €26,719 per QALY gained. Despite this very conservative scenario (which does not favor the assessed treatment), EBR/GZR is cost-effective for the studied population.

### Mortality

The combined HCV-CKD model used ESLD mortality, ESRD mortality, and other causes mortality. To take into account the rising risk over time of progressing to end-stage CKD states or of dying from chronic hepatitis C for CKD patients, baseline probabilities in the model were adjusted to the progression of CKD and mortality not specific to chronic hepatitis C or chronic kidney disease (all causes mortality) using hazard ratios obtained by comparing the CKD progression and mortality rates between CKD patients with HCV and CKD patients without HCV. Data sources for specific mortality and all causes mortality are defined in Tables 3 and 4.

### Costs

Costs were estimated for both liver and kidney diseases, by health sate. Costs (inpatient, outpatient, and pharmaceutical) for chronic liver disease patients are shown in Table 6.

**Table 6:** Costs for Natural History Health States, chronic liver disease, in Euro 2015.

Health State	Value for base case analysis (€2015)	Source / Reference
SVR, F0-F4	€0	Assumption
F0-F2	€373	[28]
F3	€431	[28]
F4	€1,560	[28]
Decompensated cirrhosis (1st year)*	€8,664	[28]
Decompensated cirrhosis (subsequent years)*	€15,786	[28]
Decompensated cirrhosis (weighted annual mean cost for first year and subsequent years)	€13,008	[28], calculation
Hepatocellular carcinoma (1st year)*	€12,289	[28]
Hepatocellular carcinoma (subsequent years)*	€12,289	[28]
Hepatocellular carcinoma (weighted annual mean cost for first year and subsequent years)	€12,289	[28]
Liver transplantation (1st year)*	€56,243	[28]
Liver transplantation (subsequent years)*	€5,846	[28]
Liver transplantation (weighted annual mean cost for 1st year and subsequent years)	€13,910	[28], calculation*
Cost of death from liver disease		
Decompensated Cirrhosis death during the 1st year)	€3,095	[28]
Decompensated Cirrhosis (death during the subsequent years)	€4,819	[28]
Hepatocellular Carcinoma (death during the 1st year)	€5,096	[28]
Hepatocellular Carcinoma (death during the subsequent years)	€5,096	[28]
Liver Transplant (death during the 1st year)	€35,016	[28]
Liver Transplant (death during the subsequent years)	€10,475	[28]

*These cost data allowed estimating the weighted annual mean costs for DC, HCC, and LT 1st year and DC, HCC, and LT following years. The calculation is as follow: Weighted annual mean cost for DC/HCC/LT = annual cost for DC/HCC/LT 1st year x annual transition probability from DC/HCC/LT 1st year to death + annual cost for DC/HCC/LT following years x (1—annual transition probability from DC/HCC/LT 1st year to death).

Costs of managing CKD patients by stage are shown in Table 7.

**Table 7:** Costs for Natural History Health States, chronic kidney disease, in Euro 2015.

CKD stage	Annual Cost (in Euro 2015) Mean (CI95% lower bound; CI95% upper bound)*	Source / Reference
CKD stage 4	€546 (€474; €617)	Calculation
CKD stage 5	€545.52 (€474.37; €616.66)	Calculation
CKD stage 5 with dialysis	€85,337 (€64,003; €106,671)	HAS [29]
Kidney transplant 1st year*	€85,079 (€63,809; €106,349)	HAS [29]
Kidney transplant following years*	€19,823 (€14,867; €24,778)	HAS [29]
Kidney transplant (weighted annual mean cost for first year and subsequent years)	€21,688 (€19,823; €85,079)	HAS [29], calculation*

* These cost data allowed estimating the weighted annual mean costs for KT. The calculation is as follow: Weighted annual mean cost for KT = annual cost for KT 1st year x annual transition probability from KT 1st year to death + annual cost for KT following years x (1—annual transition probability from KT 1st year to death).

### Model analysis

The model was run for each of the specified patient profile. An overall weighted average result was generated based on distribution of the patient characteristics. The baseline discount rate was 4%.

We calculated lifetime risk of liver disease complications, life expectancy in discounted years, discounted treatment costs, discounted health state costs, and discounted QALYs. We applied within-cycle correction to all cumulative outcomes using Simpson’s 1/3rd rule and tested results sensitivity to the correction method by applying the standard application of half-cycle correction method [33]. For the cost-effectiveness analysis, we calculated costs and QALYs over the remaining duration of a patient’s lifetime. The incremental cost-utility ratio (ICUR) of EBR/GZR regimen relative to a no treatment was calculated by dividing incremental total discounted costs by incremental total discounted number of QALYs.

#### Base-case analysis

Aggregated results are presented for genotype 1 patients and generalized for genotype 4 patients (see Conclusion).

#### Subgroup analysis

Results are also provided separately for each fibrosis stage. The robustness of these results was tested by changing baseline demographic characteristics such as sex (i.e., men only and women only) and age distribution.

#### Deterministic sensitivity analysis

We conducted one-way sensitivity analyses for several parameters showing the effect of varying these inputs on the ICUR of EBR/GZR treatment strategies compared with standard of care (SoC). We varied progression rates, efficacy, unit costs, utility weights; discount rates using ranges defined in S1 Table (cf. Supporting Information).

#### Probabilistic sensitivity analysis

In order to quantify the impact of parameter uncertainty for transition probabilities, SVR, costs and utility weights on the ICUR of EBR/GZR treatment strategy compared with no treatment, we performed probabilistic sensitivity analysis (PSA). Using Monte Carlo simulations methods, we drew 1,000 random samples from pre-defined distributions (S2 Table).

Parameters of the Gamma and Beta distributions were estimated using the method of moments that relates each parameter to the mean and standard deviation. We used the base-case values as estimates of the mean. Standard errors were estimated from confidence intervals or ranges.

Results of the PSA were summarized using descriptive statistics and cost-effectiveness acceptability curves (CEAC) [21]. The CEAC summarizes uncertainties of the cost-effectiveness results showing the probability a regimen is cost effective as a function of willing-to-pay for a QALY gained.

### Model predictions and validation

The model face validity was checked by comparing its structure to previous published models [8, 23,24]. Several tests were built into the model for verification and to ensure internal validity. We cross-validated the model by comparing its prediction of a 20-year probability of compensated cirrhosis to previous models [34,35]. These models predicted the 20-year probability of compensated cirrhosis among untreated 44-years-old patients with mild chronic HCV between 27% and 29%. Assuming that the respective distribution of mild HCV between F0 and F1 is 35% and 65%, the model projected the 20-year probability of compensated cirrhosis at 23.9%.

## Results

### Base-case analysis

The discounted results of life years and QALYs considering the lifetime simulation are presented in Table 8.

**Table 8:** Discounted life years and QALYs for 1 patient.

Strategy	Life years	QALYs
EBR/GZR regimen	6.28	6.20
Comparator: Standard of Care (no treatment)	5.09	3.73
Gain (+) or Loss (-)	+1.19	+2.47

The total number of QALYs was higher for elbasvir-grazoprevir because of a better utility on HCV health states and of a lower all causes mortality raised by complications related to hepatic and renal insufficiencies.

The other clinically important results simulated by the model are presented in Table 9.

**Table 9:** Other discounted results simulated by the model, all patients.

Clinical criteria	Elbasvir-grazoprevir (1)	Standard of Care (no treatment) (2)	Difference (1)-(2)
HCV disease-related mortality (%)	0.07%	8.67%	-8.59%
CKD disease-related mortality (%)	57.75%	50.57%	+7.18%
Other causes mortality (%)	42.18%	40.76%	+1.41%
Patients with kidney transplant (%)	21.17%	19.78%	+1.39%
Patients with decompensated cirrhosis (%)	0.85%	9.74%	-8.89%
Patients with hepatocellular carcinoma (%)	1.94%	7.85%	-5.91%
Patients with liver transplant (%)	0.04%	4.50%	-4.46%

The results of Table 9 were validated by French clinical experts, especially for HCV disease-related mortality (%), CKD disease-related mortality (%), other causes mortality (%), patients with kidney transplant (%), patients with decompensated cirrhosis (%), patients with hepatocellular carcinoma (%), and patients with liver transplant (%). The lowest mortality related to hepatic insufficiency was associated to elbasvir-grazoprevir. Patients treated with elbasvir-grazoprevir live longer (Table 9) and die later from complications related to renal insufficiency and cardiovascular diseases.

Table 10 shows the discounted cost effectiveness results. The incremental cost-utility ratio (ICUR) of elbasvir-grazoprevir regimen versus standard of care (no treatment) was €15,212 per QALY in the base case analysis.

**Table 10:** Discounted cost-effectiveness results for the base case analysis, all patients.

Strategy	Total Cost (€2015)	Life Years (LYs)	QALYs	Dominance or ICUR	
				Cost/LY (€2015)	Cost/QALY (€2015)
Standard of Care (no treatment)	€259,125	5,09	3,73	-	**-**
Elbasvir-grazoprevir	€296,672	6,28	6,20	€31,513	€15,212

### Subgroup analysis

The results of subgroup analysis are presented in Table 11.

**Table 11:** Discounted cost-effectiveness results for subgroup analysis.

Subgroup	ICUR: Cost per QALY gained (€2015)
Subgroup 1: F0 fibrosis stage and renal insufficiency stage 4–5 (with dialysis)	€15,787
Subgroup 2: F1 fibrosis stage and renal insufficiency stage 4–5 (with dialysis)	€14,948
Subgroup 3: F2 fibrosis stage and renal insufficiency stage 4–5 (with dialysis)	€13,644
Subgroup 4: F3 fibrosis stage and renal insufficiency stage 4–5 (with dialysis)	€13,721
Subgroup 5: F4 fibrosis stage (compensated cirrhosis) and renal insufficiency stage 4–5 (with dialysis)	€16,552

Results of this subgroup analysis are provided separately for each fibrosis stage (all CKD stages included). The robustness of these results was tested by changing baseline demographic characteristics such as sex (i.e., men only and women only) and age distribution. The underlying distribution of the F and CKD states of these patients is defined as follow:

Distribution of METAVIR fibrosis stages at baseline (all CKD stages included): F0: 0,223; F1: 0,241; F2: 0,071; F3: 0,214; F4: 0,250.

Distribution of CKD stages at baseline (all F stages included): CKD4: 0,300; CKD5: 0,330; CKD5-Haemodialysis (HD): 0,370; CKD5-Kidney transplant (KT): 0,000.

For this subgroup analysis, the value for F0/…/F4 was parameterized to 1, and 0 for the other F stages. This subgroup analysis was required by HAS during technical early meetings. To distribute the patients according to their concomitant F and CKD state was not possible, because of the unavailability of these data from the French HEPATHER cohort. This limitation is highlighted in the Discussion section.

### Deterministic sensitivity analysis

**Fig 3:**
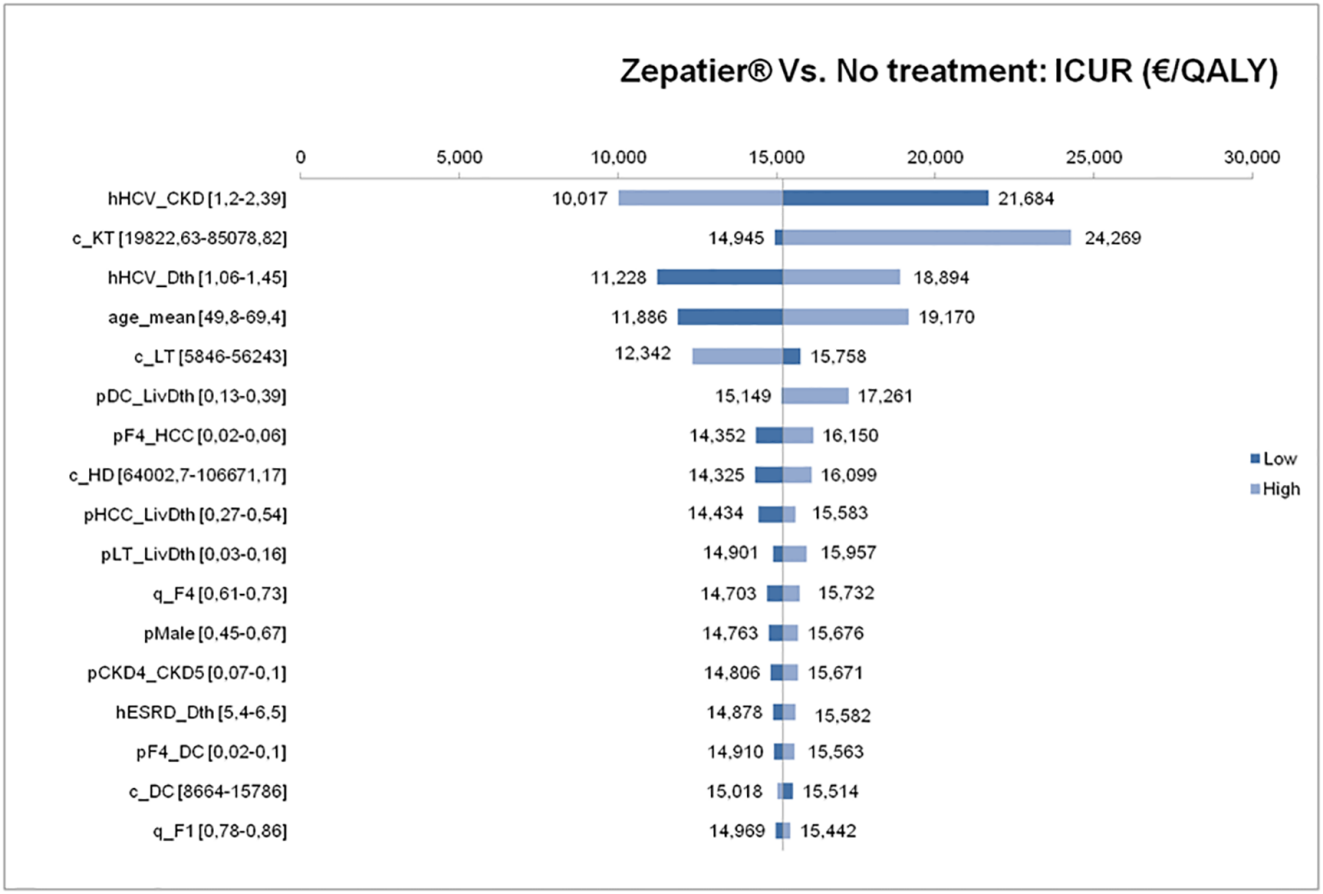
Tornado diagram: Sensitivity of input parameters on ICUR, for base case analysis. hHCV_CKD: Risk of progression of CKD given HCV (all stages), c_KT: Weighted annual mean cost for kidney transplant, hHCV_Dth: Risk of death given HCV (all stages), age_mean: Mean age of the patient, c_LT: Weighted annual mean cost for liver transplant, pDC_LivDth: Transition probability from decompensated cirrhosis to liver-related death, pF4_HCC: Transition probability from compensated cirrhosis to hepatocellular carcinoma, c_HD: Annual cost for haemodialysis, pHCC_LivDth: Transition probability from hepatocellular carcinoma to liver-related death, pLT_LivDth: Transition probability from liver transplant to liver-related death, q_F4: utility value for compensated cirrhosis, pMale: percentage Male, pCKD4_CKD5: Transition probability from CKD stage 4 to CKD stage 5, hESRD_Dth: Risk of death given end-stage renal disease, pF4_DC: Transition probability from compensated cirrhosis to decompensated cirrhosis, c_DC: Weighted annual mean cost for decompensated cirrhosis, q_F1: utility value for F1 fibrosis stage.

The tornado diagram (Fig 3) shows the ranking of input parameters according to their sensitivity on the ICUR.

The result was most sensitive to the variation of the CKD progression risk (hazard rate) given HCV (all stages). The ICUR varied of approximately €14,252, when accounting from a hazard rate of CKD progression given HCV of 2.39 (ICUR of €10,017) to a weighted annual mean cost for kidney transplant of €85,079 (ICUR of €24,269). The next three more influential parameters were the hazard rate of death given HCV (all stages), the mean age of the patient at the beginning of the simulation, and the weighted annual mean cost for liver transplant. However, the variation in ICUR obtained by changing these three parameters was less pronounced than for the two first parameters (differences between upper and lower limits of nearly €7,700, €7,300 and €3,400, respectively).

### Probabilistic sensitivity analysis

**Fig 4:**
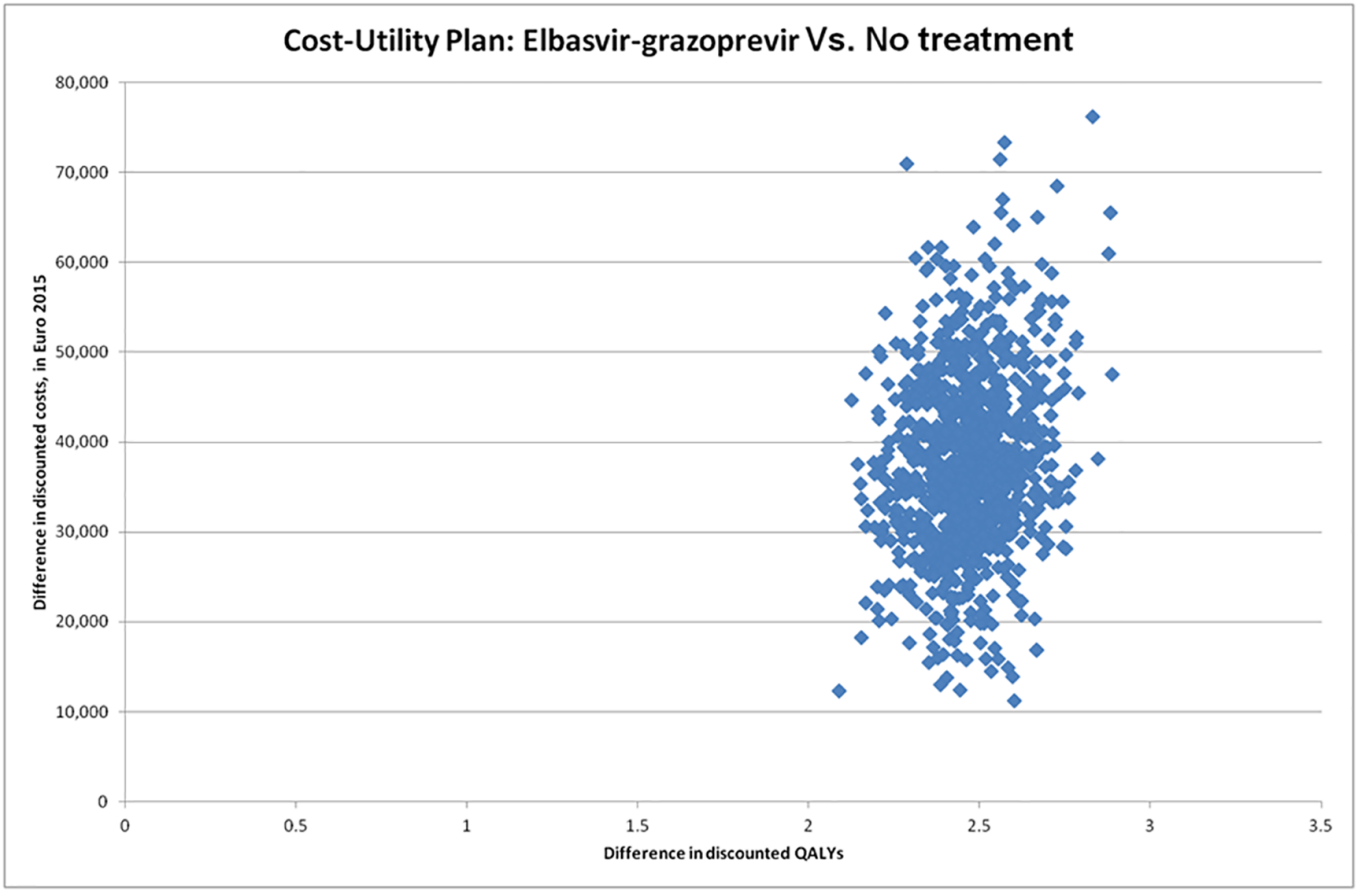
Probabilistic sensitivity analysis: Cost-Utility plan, for the base case analysis.

The PSA cost-effectiveness plane (Fig 4) describes the difference in effectiveness (QALYs) on the x-axis and the difference in cost (Euro 2015) on the y-axis between the two compared strategies, for 1,000 simulations in each group.

**Fig 5:**
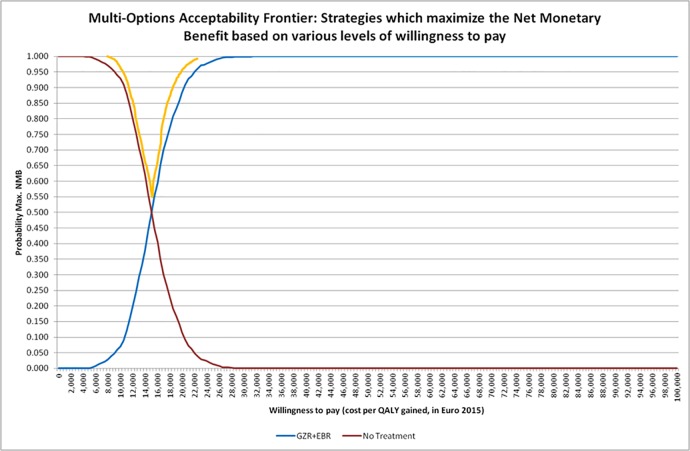
Probabilistic sensitivity analysis (PSA): Cost-Utility acceptability curves (CUAC, blue and red curves) and multi-options acceptability frontier (orange frontier). The NMB is defined as the monetary value of the health benefit in each strategy, expressed as follow: NMB = Lambda x Mean Effectiveness (QALY)–Mean Cost, with lambda depicting various values of willingness to pay.

100% of points (ICUR) of the simulation were located in the North-East quadrant of the cost-effectiveness plane. The North-East quadrant is the era where EBR/GZR is more effective and more costly than the comparator. So, the ICUR expressed as Euro per QALY has to be estimate. The acceptability curves (Fig 4) shows that the probability of being cost-effective for the assessed strategy was higher than the comparator (no treatment) starting from a willingness to pay of €15,300 per QALY gained (probability of 50%). For gaining 30% of confidence (from 50% to 80%), the willingness to pay by the community passes from €15,300 to less than €18,500. The ICUR values corresponding to 70% and 80% of simulations (% of cost-effective results on 1000 simulations) were respectively €17,000 and €18,300. To reach the asymptote with 100% of simulations, the ICUR value was of €31,500. The multi-options acceptability frontier (Fig 5) presents the border (in orange) of the interventions which maximize the net monetary benefit (NMB) according to various levels of willingness to pay by the community to gain a QALY.

The strategy that maximized the net monetary benefit up to a willingness to pay less than € 15,000 (lambda) was the reference strategy (no treatment). Beyond this level of willingness to pay to win a QALY, the assessed strategy elbasvir-grazoprevir maximized the net monetary benefit.

## Discussion

This cost-utility model is, to our knowledge, the first considering the evolution of both chronic hepatitis C and chronic kidney disease when HAS assessment has been done. We found that using elbasvir-grazoprevir to treat HCV infected patients with chronic kidney disease was cost effective for payers willing to pay above €15,000/QALY. The treatment was increasingly efficient in the more advanced stages of the renal insufficiency.

Considering the objective of public health it is thus important to treat HCV-CKD patients as early as possible. As the CKD progression was considered we obtained a conservative result of cost-effectiveness in HCV patients, because patients treated by elbasvir-grazoprevir live longer than untreated patients and thus have a raising risk over time to develop some complications related to chronic kidney disease. As a consequence, to take into account the renal insufficiency progression in the base case analysis would make it possible to consider this scenario as a conservative scenario.

A paper published by Elbasha *et al*. (December, 2016) [36] considered the same objective of our study but in different settings (US settings). The ICUR of EBR/GZR compared with No Treatment was of $13 200/QALY, which is very similar to our findings, despite significant differences in Health care settings.

Regarding the availability of treatment comparators, no available information has been found for Glecaprevir /Pibrentasvir. Glecaprevir/Pibrentasvir is Abbvie’s treatment for which we had no information at the time of the HAS assessment. We have considered whether the AbbVie Phase III clinical trial could have been considered in relation to the population we are modeling. Unfortunately, we had methodological limitations preventing to include this regimen into a network meta-analysis to take it into account in our cost-utility study. Indeed, the web link (Last Update Posted: February 2, 2018) https://clinicaltrials.gov/ct2/show/NCT03069365 states that “No Study Results Posted on ClinicalTrials.gov for this Study” (access online: 2018/02/19). In addition, the study design can be considered having limitations such as non-randomization and non-masking. Moreover, the comparator of the above study is not defined.

For all these reasons we could not include this treatment in the analysis.

Transition probabilities are mainly taken from the published paper of Deuffic-Burban et al. 2014 [28] which is very suited to French settings for the simulated population. This published study has been validated by HAS for populating the French cost-utility study. Potential differences in transition probabilities in comparison to other models [36] are taken into account through deterministic and probabilistic sensitivity analyses, which consider ranges of values for each transition probability and subsequent variation of ICUR highlighted by the tornado chart (Fig 3), the Cost-Utility plane (Fig 4), and the multi-options acceptability frontier (Fig 5).

For the “Risk of progression of CKD given HCV (all stages)” in the current model, a hazard rate of 1.7 is used. This hazard rate is taken from the published meta-analysis of Fabrizi et al. 2014 [30]. This source has been validated by the scientific board of the cost-utility study for the following reasons: This is a meta-analysis of 7 longitudinal observational studies including 890,560 patients with chronic hepatitis C and severe end-stage chronic renal failure. The objective of this study was to estimate the statistical relationship, measured by hazard ratios (HR), between HCV seropositivity and the reduction in estimated glomerular filtration rate (eDFG), which is a marker deterioration of renal function. The results of HR are not available for each stage of liver fibrosis, but in all stages. The adjusted mean HR is estimated at 1.70 with a 95% confidence interval between 1.20 and 2.39. Thus, it is considered in the base case analysis of the model that the presence of chronic hepatitis C in patients with renal insufficiency increases the risk of progression from severe renal insufficiency (stage 4) to end-stage renal failure (stage 5), and from end stage renal failure to hemodialysis, of 1.70, compared to the natural course of renal failure without hepatitis C. The value of 1.32 published for the US model [36] is included in the 95% CI values which were taken into account in deterministic and probabilistic sensitivity analyses.

The assumption that there are no costs of care in SVR F0 to F4 could be challenged. Most patients, especially those with F2, F3 and F4 liver damage could continue to require health care. For testing this assumption, we performed a scenario analysis considering same values for SVR F0 to F4 costs and F0 to F4 costs (without SVR), for each fibrosis stage (annual cost of 373 € for SVR-F0/F1/F2, 431 € for SVR-F3, and 1 560 € for SVR-F4). This scenario is very conservative as it considers that SVR has no impact on annual costs of care. For this scenario, the ICUR increases at €16,772 per QALY gained. Despite this very conservative scenario (which does not favor the assessed treatment), EBR/GZR is cost-effective for the studied population. On top of that, for being much more conservative, we also considered a 0.82 utility index for SVR state, whatever the fibrosis stage. This value is equal to the utility index used for F0-F1 health states. For this scenario, the ICUR increases at €29,460 per QALY gained. Despite this very concervative scenario for the product, EBR/GZR is still cost-effective for the studied population.

Some of the main limitations of our model could favor the assessed treatment, as the the absence of utility decrement for CKD. The non inclusion of potential stopping rules for HCV treatment is another limitation. The initial distribution of patients (at baseline) was considered distinctly for the liver (F0 to F4 fibrosis stages) and for the kidney disease (CKD stages 4–5 and 5 with dialysis), and the model therefore calculated the benefit of the treatment for two instead of one statistical individuals; a distribution of HCV-CKD combined health states was not available from the HEPATHER French cohort. The current CKD model structure did not make it possible for patients with CKD stage 4 to reach dialysis, but the C-Surfer clinical study [6] showed that a few CKD stage 4 patients with GFR < 20 ml/min were dialyzed; this could be considered as a non-conservative limitation. The costs related to ESLD mortality, ESRD mortality, and cardiovascular disease mortality were not taken into account, but the deterministic sensitivity analysis showed that ICUR decreased from €15,212, with a conservative null death cost, to €14,313 if the HCV-related death cost was of €35,016 (i.e. the maximum cost based on the literature [28]). The treatment cost related to the last month when patient deceased [29] was of approximately €10,799 (standard deviation: €10,075) in CKD stage 5 patient (GFR < 15 ml/min/1,73m2) treated with hemodialysis. The potential limitation of considering a null death cost related to ESRD was thus balanced by the conservative approach suggested into the base case analysis which also did not consider a death cost due to ESLD. Indeed, the Markov states “ESLD Mortality”, “ESRD Mortality”, and “Other-causes Mortality” are absorbent states and valued to zero cost, for being considered as a recommended scenario by HAS.

In France, the reimbursement decision is based on clinical effectiveness and not on cost-effectiveness outcomes. Clinical effectiveness and cost-effectiveness are assessed independently and in parallel by different committees. The HAS, in giving its efficiency opinion, does not make recommendations about reimbursement. The economic evaluation is one among other criteria (e.g. added clinical benefit) that is used by the French Pricing Committee (CEPS) for negotiating the price with the manufacturers. Since January 2016, in addition to the cost-effectiveness analysis (CEA) submissions, budget impact analyses (BIAs) are required as part of efficiency dossiers submitted by manufacturers for innovative drugs with an expected 2-year sales revenue above €50 million. For EBR/GZR dossier, the expected 2-year sales revenue in the studied population is below €50 million; thereby, a BIA was not required by HAS.

## Conclusions

Treatment with elbasvir-grazoprevir was found to be cost effective in HCV-genotypes 1/4 and CKD-stage 4–5 or 5 with dialysis patients. With a treatment cost of €29,402 for 12 weeks of treatment, 100% of simulations were below €31,500 per QALY gained.

This study has the non-technical limitation of being sponsored by industry (the MSD Company). However, an external research organization (Statesia) was hired to handle independently the adaptation of the simulation model and the data analysis to remove any possible bias.

## Supporting information

S1 File Markov traces of the cost-effectiveness model for base case analysis. (XLSX)

S2 File Input data and hypotheses of the cost-effectiveness model for base case analysis. (XLSX)

S1 Table One way deterministic sensitivity analysis. ^**†**^For a few cases, only one value can be tested. *Non-CKD: Comparable patients with GFR ≥ 60 ml/min/1,73 m^2^ (HR = 1). HR, Hazard Rate.
(DOCX)

S2 Table Probabilistic sensitivity analysis: Distributions and parameter values. (DOCX)
