# Supplementary material for: Cost-effectiveness analysis of elbasvir-grazoprevir regimen for treating hepatitis C virus genotype 1 infection in stage 4-5 chronic kidney disease patients in France
Source: PLoS One. 2018 Mar 15;13(3):e0194329. doi: 10.1371/journal.pone.0194329 (PMC5854359; doi:10.1371/journal.pone.0194329)
Supplement: S1 Table — †For a few cases, only one value can be tested. *Non-CKD: Comparable patients with GFR ≥ 60 ml/min/1,73 m2 (HR = 1). HR, Hazard Rate. (DOCX) [file pone.0194329.s003.docx]

**S1 Table: One Way Deterministic Sensitivity Analysis**

| **Value for base case analysis** | **Sensitivity analysis [lower limit - upper limit]✝** | **Source/Justification** |
| --- | --- | --- |
| Mean age (59.6 years) | 49.8 years and 69.4 years | Hepather [calculation / standard error: 9.8 years] |
| % Men (56%) | 45% and 67% | [- 20% ; +20%] arbitrary |
| SVR rates EBR/GZR (99.1%) | 95% and 100% | C-Surfer [6] |
| Annual transition probabilities | | |
| F0 to F1 (0.072) | 0.068 and 0.076 | [25] |
| F1 to F2 (0.101) | 0.098 and 0.103 | [25] |
| F2 to F3 (0.108) | 0.106 and 0.111 | [25] |
| F3 to F4 (0.210) | 0.206 and 0.213 | [25] |
| F4 to DC (0.050) | 0.017 and 0.100 | [25] |
| F4 to HCC (0.036) | 0.020 and 0.056 | [25] |
| DC to HCC (0.036) | 0.020 and 0.056 | [25] |
| DC to LT (0.120) | 0.097 and 0.145 | [25] |
| HCC to LT (0.170) | 0.143 and 0.199 | [25] |
| DC to HCV-related death (0.130) | 0.125 and 0.390 | [27] / [25] |
| HCC to HCV-related death (0.430) | 0.270 and 0.540 | [27] / [25] |
| LT to HCV-related death (0.060) | 0.032 and 0.160 | [27] / [25] |
| SVR F4 to DC (0.004) | 0.002 and 0.011 | [25] |
| SVR F4 to HCC (0.010) | 0.005 and 0.011 | [25] |
| CKD4 to CKD5 (0.081) | 0.067 and 0.096 | [25] |
| CKD5 to CKD5 dialysis (0.434) | 0.430 and 0.439 | [17] |
| CKD5 to KT (0.035) | 0.034 and 0.037 | [17] |
| CKD5 dialysis to KT (0.048) | 0.046 and 0.050 | [17] |
| KT to CKD5 dialysis (0.086) | 0.084 and 0.088 | [17] |
| CKD5 to death (0.070) | 0.068 and 0.072 | [17] |
| CKD5 dialysis to death (0.125) | 0.123 and 0.128 | [17] |
| KT to death (0.029) | 0.027 and 0.030 | [17] |
| HR Stroke/MI CKD4 vs. NO CKD* (2.80) | 2.60 and 2.90 | [29] |
| HR Stroke/MI CKD5 vs. NO CKD* (3.40) | 3.10 and 3.80 | [29] |
| HR all-cause mortality CKD4 vs. NO CKD* (3.20) | 3.10 and 3.40 | [29] |
| HR all-cause mortality CKD5, CKD5 dialysis vs. NO CKD* (5.90) | 5.40 and 6.50 | [29] |
| HR CKD progression given HCV (all stages) vs. NO HCV (1.70) | 1.20 and 2.40 | [31] |
| HR mortality given HCV (all stages) vs. NO HCV (1.24) | 1.06 and 1.45 | Calculation |
| Annual Costs (in Euro 2015) | | |
| F0 (€373) | €280 and €466 | [25] / [- 25% ; +25%] arbitrary |
| F1 (€373) | €280 and €466 | [25] / [- 25% ; +25%] arbitrary |
| F2 (€373) | €280 and €466 | [25] / [- 25% ; +25%] arbitrary |
| F3 (€431) | €323 and €538 | [25] / [- 25% ; +25%] arbitrary |
| F4 (€1,560) | €1 170 and €1 950 | [25] / [- 25% ; +25%] arbitrary |
| Weighted annual mean cost for DC first year and DC following years (€13,008) | €8,664 and €15,786 | Calculation / [25] |
| Weighted annual mean cost for HCC first year and HCC following years (€12,289) | €9,217 and €15,362 | Calculation / [25] |
| Weighted annual mean cost for LT first year and LT following years (€13,910) | €5,846 and €56,243 | Calculation / [25] |
| ESLD-related death cost (conservative value: 0 €) | 0 and €35,016 | [25] / Conservative assumption |
| Weighted annual mean cost for KT first year and KT following years (€21,688) | €19,823 and €85,079 | Calculation / [17] |
| Annual cost per CKD4 patient (€546) | €474 and €617 | [32] / Calculation |
| Annual cost per CKD5 patient (€1,324) | €1,012 and €1,632 | [32] / Calculation |
| Annual cost for CKD5 patient with dialysis (€85,337) | €64,003 and €106,671 | [17] / [- 25% ; +25%] arbitrary |
| Discount rate for costs and health outcomes (4%) | 0% and 6% (2.5%) | [8] /[27] |
| Utility Values | | |
| Utility during EBR/GZR treatment (1.00) | 0.95 | C SURFER [3], According to medical experts / -5% |
| Utility after treatment, F0 (0.82) | 0.78 and 0.86 | [30] / Standard error = 0,02 |
| Utility after treatment, F1 (0.82) | 0.78 and 0.86 | [30] / Standard error = 0,02 |
| Utility after treatment, F2 (0.78) | 0.74 and 0.82 | [30] / Standard error = 0,02 |
| Utility after treatment, F3 (0.67) | 0.61 and 0.73 | [30] / Standard error = 0,03 |
| Utility after treatment, F4 (0.67) | 0.61 and 0.73 | [30] / Standard error = 0,03 |
| Utility after treatment, weighted mean DC 1st year and subsequent years (0.51) | 0.37 and 0.65 | [30] / Standard error = 0,07 |
| Utility after treatment, weighted mean HCC 1st year and subsequent years (0.51) | 0.37 and 0.65 | [30] / Standard error = 0,07 |
| Utility after treatment, weighted mean LT 1st year and subsequent years (0.75)*** | 0.53 and 0.91 | [30] / Standard error = 0,10 |
| Utility after treatment, LT following years (0.80) | 0.62 and 0.93 | [30] / Standard error = 0,08 |

^✝^For a few cases, only one value can be tested.

*Non-CKD: Comparable patients with GFR ≥ 60 ml/min/1,73 m² (HR=1).

HR, Hazard Rate.
