# Supplementary material for: Cost-effectiveness analysis of elbasvir-grazoprevir regimen for treating hepatitis C virus genotype 1 infection in stage 4-5 chronic kidney disease patients in France
Source: PLoS One. 2018 Mar 15;13(3):e0194329. doi: 10.1371/journal.pone.0194329 (PMC5854359; doi:10.1371/journal.pone.0194329)
Supplement: S2 Table — (DOCX) [file pone.0194329.s004.docx]

**S2 Table: Probabilistic Sensitivity Analysis: Distributions and parameter values**

| **Variable / Input Parameter** | **Distribution** | **Standard error** | **Distribution Parameter 1** | **Distribution Parameter 2** |
| --- | --- | --- | --- | --- |
| SVR rates | Beta | 0.013 | 51.09 | 0.44 |
| Annual probability F0 to F1 (0.072) | Beta | 0.002 | 1,081.14 | 13,989.09 |
| Annual probability F1 to F2 (0.101) | Beta | 0.001 | 4,776.99 | 42,717.44 |
| Annual probability F2 to F3 (0.108) | Beta | 0.001 | 6,085.05 | 50,091.60 |
| Annual probability F3 to F4 (0.210) | Beta | 0.002 | 13,307.42 | 50,091.42 |
| Annual probability F4 to DC (0.050) | Beta | 0.021 | 5.10 | 96.90 |
| Annual probability F4 to HCC (0.36) | Beta | 0.009 | 14.99 | 400.01 |
| Annual probability DC to HCC (0.036) | Beta | 0.009 | 14.99 | 400.01 |
| Annual probability DC to LT (0.120) | Beta | 0.012 | 83.88 | 615.12 |
| Annual probability HCC to LT (0.170) | Beta | 0.014 | 118.83 | 580.17 |
| Annual probability DC HCV-related death (0.130) | Beta | 0.068 | 3.09 | 20.66 |
| Annual probability HCC HCV-related death (0.430) | Beta | 0.019 | 300.57 | 398.43 |
| Annual probability LT HCV-related death (0.060) | Beta | 0.004 | 178.08 | 2,789.92 |
| Annual probability SVR F4 to DC (0.004) | Beta | 0.002 | 3.02 | 751.78 |
| Annual probability SVR F4 to HCC (0.010) | Beta | 0.001 | 43.43 | 4,409.75 |
| Annual probability CKD4 to CKD5 (0.081) | Beta | 0.007 | 110.09 | 1,249.03 |
| Annual probability CKD5 to CKD5 with dialysis (0.434) | Beta | 0.002 | 17,805.64 | 23,176.36 |
| Annual probability CKD5 to KT (0.035) | Beta | 0.001 | 2,224.98 | 61,171.02 |
| Annual probability CKD5 dialysis to KT (0.048) | Beta | 0.001 | 3,422.30 | 67,875.70 |
| Annual probability KT to CKD5 dialysis (0.086) | Beta | 0.001 | 6,136.53 | 65,161.47 |
| Annual probability CKD5 to death (0.070) | Beta | 0.001 | 4,437.72 | 58,958.28 |
| Annual probability CKD5 dialysis to death (0.125) | Beta | 0.001 | 8941.48 | 62,356.52 |
| Annual probability KT to death (0.029) | Beta | 0.001 | 2,037.95 | 69,260.05 |
| HR Stroke/MI CKD4 vs. NO CKD* (2.80) | LogNormal | 0.028 | 1.030 | 0.028 |
| HR Stroke/MI CKD5 vs. NO CKD* (3.40) | LogNormal | 0.052 | 1.224 | 0.052 |
| HR CKD 4 all-cause mortality vs. NO CKD* (3.20) | LogNormal | 0.024 | 1.163 | 0.024 |
| HR CKD5-5 dialysis all-cause mortality vs. NO CKD* (5.90) | LogNormal | 0.047 | 1.775 | 0.047 |
| HR CKD Progression given HCV (all stages) vs. NO HCV (1.70) | LogNormal | 0.176 | 0.531 | 0.176 |
| HR mortality given HCV (all stages) vs. NO HCV (1.24) | LogNormal | 0.080 | 0.215 | 0.080 |
| Annual cost F0 state (€373) | Gamma | 48 | 61.47 | 6.06 |
| Annual cost F1 state (€373) | Gamma | 48 | 61.47 | 6.06 |
| Annual cost F2 state (€373) | Gamma | 48 | 61.47 | 6.06 |
| Annual cost F3 state (€431) | Gamma | 55 | 61.47 | 7.01 |
| Annual cost F4 state (€1,560) | Gamma | 199 | 61.47 | 25.38 |
| Mean annual cost DC state (€13,008) | Gamma | 1,659 | 61.47 | 211.63 |
| Mean annual cost HCC state (€12,289) | Gamma | 1,568 | 61.47 | 199.94 |
| Mean annual cost LT state (€13,910) | Gamma | 1,774 | 61.47 | 226.30 |
| Mean annual cost for kidney transplant KT (€21,688) | Gamma | 2,766 | 61.47 | 352.85 |
| Annual cost for CKD4 patient (€546) | Gamma | 70 | 61.47 | 8.88 |
| Annual cost for CKD5 patient (€1,324) | Gamma | 169 | 61.47 | 21.54 |
| Annual cost for CKD5 patient with dialysis (€85,337) | Gamma | 10,885 | 61.47 | 1,388.37 |
| Utility after treatment, F0 state (0.82) | Beta | 0.020 | 301.76 | 66.24 |
| Utility after treatment, F1 state (0.82) | Beta | 0.020 | 201.76 | 66.24 |
| Utility after treatment, F2 state (0.78) | Beta | 0.020 | 333.84 | 94.16 |
| Utility after treatment, F3 state (0.67) | Beta | 0.030 | 163.93 | 80.74 |
| Utility after treatment, F4 state (0.67) | Beta | 0.030 | 163.93 | 80.74 |
| Utility after treatment, DC state 1st year (0.51) | Beta | 0.070 | 25.50 | 24.50 |
| Utility after treatment, DC state following years (0.51) | Beta | 0.070 | 25.50 | 24.50 |
| Utility after treatment, average DC state (0.51) | Beta | 0.070 | 25.50 | 24.50 |
| Utility after treatment, HCC state 1st year (0.51) | Beta | 0.070 | 25.50 | 24.50 |
| Utility after treatment, HCC state following years (0.51) | Beta | 0.070 | 25.50 | 24.50 |
| Utility after treatment, average HCC state (0.51) | Beta | 0.070 | 25.50 | 24.50 |
| Utility after treatment, LT state 1st year (0.46) | Beta | 0.100 | 10.97 | 12.87 |
| Utility after treatment LT state following years (0.80) | Beta | 0.080 | 19.20 | 4.80 |
| Utility after treatment, average LT state (0.75) | Beta | 0.10 | 13.40 | 4.57 |
